# Supplementary material for: Optimising Regimen of Co-Amoxiclav (ORCA)—The Safety and Efficacy of Intravenous Co-Amoxiclav at Higher Dosing Frequency in Patients with Diabetic Foot Infection
Source: Antibiotics (Basel). 2025 Jul 28;14(8):758. doi: 10.3390/antibiotics14080758 (PMC12382847; doi:10.3390/antibiotics14080758)
Supplement: Supplementary file 1 [file antibiotics-14-00758-s001.zip › antibiotics-3715387-supplementary.pdf]

Supplementary Table S1. IWGDF/IDSA classification on severity of DFI.

Clinical Classification of Infection	IWGDF/IDSA Classification
No systemic or local symptoms or signs of infection	1 / Uninfected
⩾2 items present:<br>• Local swelling or induration<br>• Erythema > 0.5 but < 2 cm around the wound<br>• Local tenderness or pain<br>• Local increased warmth<br>• Purulent discharge<br>**AND** no other cause of an inflammatory response of skin	2 / Mild
Infection with no systemic manifestations and involving:<br>• Erythema extending ⩾ 2 cm from the wound margin **AND/OR**<br>• Tissue deeper than skin and subcutaneous tissues (tendon, muscle, joint, and bone)	3 / Moderate<br>Add "(O)" if osteomyelitis
Any foot infection with associated systemic manifestations (SIRS):<br>⩾ 2 of the following:<br>• Temperature > 38˚C or < 36˚C<br>• HR > 90 beats / minute<br>• RR > 20 breaths / minute<br>• WBC >12g/L **OR** < 4g/L	4 / Severe<br>Add "(O)" if osteomyelitis

Abbreviations: *DFI* Diabetic Foot Infection, *HR* Heart Rate, *IDSA* Infectious Diseases Society of America, *IWGDF* International Working Group on the Diabetic Foot, *RR* Respiratory Rate, *SIRS* Systemic Inflammatory Response Syndrome, and *WBC* White Blood Cell.

Supplementary Table S2. Factors when determining clinical deterioration at end of IVCA therapy.

Clinical Response	Factors to Determine Clinical Deterioration [£¥]
**Wound Condition**	Any sign that suggests wound condition is worsening during wound inspection, wound debridement, or wound dressing at bedside or surgery, as compared to baseline wound inspection during admission at the emergency department. These signs include:<br>- New development of findings or increased percentage/volume of findings following wound inspection (findings refer to necrosis, gangrene, slough, and pus)<br>- Extension by length, area, or depth of wound compared to previous wound inspection<br>- New development of foul smell or increased foot wound odor<br>- New development of DFO
**SIRS criteria**	⩾ 2 of the following:<br>- Temperature > 38˚C or < 36˚C<br>- HR > 90 beats / minute<br>- RR > 20 breaths / minute<br>- WBC >12g/L **OR** < 4g/L
**Inflammatory Markers**	Any sign that suggests patient has worsening inflammation or infection of the lower limb. These signs include:<br>- Up trending CRP of at least 2 values<br>- Up trending procalcitonin of at least 2 values; second value > 0.5μg/L<br>- Persistent leukocytosis with up trending WBC of at least 2 values; second value > 10g/L

Abbreviations: *CRP* C-Reactive Protein, *DFO* Diabetic Foot Osteomyelitis, *HR* Heart Rate, *IVCA* Intravenous Co-Amoxiclav, *RR* Respiratory Rate, *SIRS* Systemic Inflammatory Response Syndrome, and *WBC* White Blood Cell.

£ For patients to be classified as “clinically deteriorated”, they must have worsened wound condition, in combination with either signs that were suggestive of worsening sepsis or worsening inflammatory markers or both.

¥ In the event of clinical deterioration, primary care physicians may choose to adopt “culture-directed change” (via broadening the coverage of microorganisms based on culture results) or adopt “empirical escalation” (due to lack of clinical improvement while on IVCA therapy, prior to the release of relevant lower limb culture results).
